# Desperate Prawns: Drivers of Behavioural Innovation Vary across Social Contexts in Rock Pool Crustaceans

**DOI:** 10.1371/journal.pone.0139050

**Published:** 2015-10-21

**Authors:** Callum Duffield, Alastair J. Wilson, Alex Thornton

**Affiliations:** Centre for Ecology and Conservation, University of Exeter, Penryn Campus, Cornwall, United Kingdom; Liverpool John Moores University, UNITED KINGDOM

## Abstract

Innovative behaviour may allow animals to cope with changes in their environment. Innovative propensities are known to vary widely both between and within species, and a growing body of research has begun to examine the factors that drive individuals to innovate. Evidence suggests that individuals are commonly driven to innovate by necessity; for instance by hunger or because they are physically unable to outcompete others for access to resources. However, it is not known whether the factors that drive individuals to innovate are stable across contexts. We examined contextual variation in the drivers of innovation in rock pool prawns (*Palaemon spp*), invertebrates that face widely fluctuating environments and may, through the actions of tides and waves, find themselves isolated or in groups. Using two novel foraging tasks, we examined the effects of body size and hunger in prawns tested in solitary and group contexts. When tested alone, small prawns were significantly more likely to succeed in a spatial task, and faster to reach the food in a manipulation task, while hunger state had no effect. In contrast, size had no effect when prawns were tested in groups, but food-deprived individuals were disproportionately likely to innovate in both tasks. We suggest that contextual variation in the drivers of innovation is likely to be common in animals living in variable environments, and may best be understood by considering variation in the perception of relative risks and rewards under different conditions.

## Introduction

Animals living in variable environments may encounter novel problems, for instance as new resources become available or previously common resources become less accessible. One way individuals may overcome such challenges is through behavioural innovation, defined as “the creation of new behaviour patterns, or the performance of established behaviour patterns in a novel context” [1]. Anecdotal reports of novel foraging behaviours in birds and mammals indicate that innovative propensities vary widely between species and correlational evidence suggests that high rates of innovation may facilitate niche expansion and resilience to environmental change [2–5]. It is also increasingly clear that there is substantial variation within species in the tendency of different individuals to innovate, and a growing body of research has begun to explore the morphological, developmental and behavioural characteristics that drive this variation (reviewed in [6]). However, we have little understanding of whether the drivers of individual innovatory tendencies are stable across contexts. For instance, do the same factors explain which individuals innovate when alone or in groups, or in the presence or absence of predators?

Two main classes of explanation have been developed to understand between-individual variation in innovation. The “spare time” hypothesis suggests that animals tend to innovate when released from pressing time and energy constraints. This may help to explain why captive animals, which do not face the need to forage or avoid predators, often show higher rates of innovation than their wild counterparts [1,7,8]. More commonly, individual variation in innovation has been explained under the broad banner of the “necessity is the mother of invention” hypothesis. Under this view, individuals of low competitive ability may invest in behavioural innovation as a means of acquiring resources while avoiding direct competition. Accordingly, a number of studies on mammals, birds and fish have found that innovators tend to be low-ranking [9–13], young [14–16] and/or small [17]. Risk-sensitivity theory suggests that current motivational states may also have an important influence, with individuals being particularly likely to engage in performing potentially risky innovative behaviour if they are hungry or in poor condition. Laland and Reader [17] for example found that hunger, as well as size and sex had strong effects on innovatory propensities in groups of guppies (*Poecilia reticulata*), with food-deprived, small and female individuals being particularly likely to complete novel foraging tasks.

Results from experimental studies of innovation in vertebrates indicate that individuals’ assessments of current competition and risk are crucial in determining investment in innovation [6]. As such assessments are likely to be highly sensitive to variation in the physical and social environments, individual innovatory tendencies may vary substantially across contexts. Morand-Ferron et al, [14] for instance, found that the propensity of individual great tits (*Parus major*) to innovate in captivity was unrelated to their likelihood of innovating in the wild. Similarly, a number of studies point to important effects of social context on innovatory tendencies [18–20]. Griffin et al [19], for example, found that Indian mynahs (*Acridotheres tristis*) were substantially more likely to complete an innovative foraging task when tested alone rather than in groups, a finding the authors attributed to negotiation over risk within social groups. Nevertheless, although it is clear that innovation is context-dependent, it remains to be tested whether specific phenotypic or state-dependent drivers of innovative tendencies vary between contexts. For instance, one might hypothesise that small size or low competitive ability may promote innovation within social groups whereas investment in innovation by isolated individuals may be driven more by their current energetic state. Context-dependent differences in the drivers of innovation may be particularly prevalent in animals exposed to frequent environmental variation. These include species with fission-fusion structures, where the social environment can change substantially from day to day, and animals living in highly heterogeneous and temporally variable habitats.

Shorelines are amongst the most highly dynamic and variable habitats on earth, where the movements of tides and waves may deposit animals in widely differing locations, in the presence of conspecifics or alone in isolated pools. However, no study has yet examined the drivers of innovation in inter-tidal species. Moreover, despite growing interest in the behavioural flexibility and learning abilities of invertebrates [21,22], studies of innovation remain restricted to vertebrate species. In this study, we used novel foraging tests to determine the drivers of innovation across social contexts in shoreline prawns (*Palaemon* spp.), common inhabitants of the littoral zone. *Palaemon* prawns are omnivorous crustaceans, ranging in size from around 15 to 90mm [23–24]. They typically spend the winter offshore, at depths of 30–50m, and migrate inshore during the spring and summer, when they are commonly found in inter-tidal rock pools, either alone or in groups [23–26]. We compared prawns that differed in size and hunger levels in solitary and social contexts across two simple foraging tasks designed to mirror natural situations where the movement of materials may cover or block direct pathways to food sources. The spatial task (adapted from [17]) required prawns to find an indirect route to food, while the manipulation task required individuals to remove a small box obstructing their access to food. We predicted that small size would drive individuals to innovate to obtain resources in social contexts where they faced competition, whereas hunger would drive innovation when alone.

## Materials and Methods

### Subjects and Housing

All procedures were approved by the University of Exeter Biosciences Ethics committee. No specific permits were required for collection of prawns as they are common marine invertebrates and are not endangered or protected. We caught ca. 600 prawns (*Palaemon* spp.) in rock pools on public land around Flushing, Cornwall, UK using hand-held nets, placed them in a bucket filled with sea water and transported them to the laboratory within one hour (no other species were collected or disturbed). Once in the laboratory, we weighed them on precision digital scales. We kept the heaviest 160 and lightest 160 individuals for use in experiments (mean mass ± S.E. of large prawns: 1.308 ± 0.049g; small prawns: 0.168 ± 0.006g) and returned the rest to the sea. Any individual showing signs of ill health (decolouration, not feeding or moving normally) or injury was separated for up to 14 days until recovery. If it failed to recover or died, it was replaced with a healthy individual. At the end of the experiments the prawns were euthanised using tricaine mesylate (MS-222: 3g/litre of tricaine mesylate buffered with 3g/litre sodium bicarbonate) and transferred to a freezer at -18°C. The two common *Palaemon* sp. prawns found in Cornish rock pools are indistinguishable when alive, so we assigned individuals to species *post-mortem* by counting the number of teeth along the rostrum (*P*. *elegans*: 8–9 teeth along the top of the rostrum; *P*. *serratus*: 6–7 teeth and a slightly upward pointing rostrum). The great majority of prawns used in our experiments were *P*. *elegans*, with only six identified as *P*. *serratus* and five being ambiguous.

Experimental prawns were housed in sets of eight same-sized individuals in clear plastic tanks measuring 30 x 40cm containing continually filtered and aereated lab-produced salt water (salinity: 31–37 parts per thousand) at a depth of 40cm and temperature of 15.2–17.8°C, with a daily 12:12 light: dark cycle. Prawns were allowed to habituate to the tanks for two days to reduce stress and were then tagged for individual recognition using two of four different colours of visible implant elastomer (VIE) which were injected into the muscle tissue, one on each side of the body. This method has been extensively evaluated for use on prawns and shown to cause no long-term discomfort or behavioural changes [27]. Given limitations on the range of possible colour combinations, tags on prawns used for Group tasks were used to identify treatment groups, rather than specific individuals. Following tagging, prawns were given two days to recover before being used in experiments. When not taking part in experiments, prawns were fed each morning with 3g of cichlid pellets per tank

### Experimental Treatments

We tested prawns in two social conditions: Individual tests involved a single prawn, while Group tests involved 16 prawns together. To avoid any confounding effects of prior experience on social context, we used different prawns for Individual and Group tests. All sample sizes refer to numbers of prawns within each of the two social contexts. Prawns in both Individual and Group tasks were divided into four experimental treatments based on size and hunger state: (1) large and satiated; (2) large and hungry; (3) small and satiated and (4) small and hungry. Each treatment contained a total of 80 tagged individuals, housed in sets of eight. Each set was fed according to its hunger category. Satiated treatments were fed a pinch of cichlid pellets daily and immediately after each experimental trial, so as to standardise the food eaten in a trial regardless of task success. Hungry treatments were deprived of food for 24 hours before a trial but fed immediately after a behavioural trial. Each prawn was reweighed following the completion of all experiments to ensure no individuals changed size classes (13 days between first and last weighing). Within both size classes, food deprivation had a clear effect, significantly reducing levels of weight gain compared to satiated individuals (weight change of large prawns: hungry = -0.105 ± 0.039g, satiated = 0.025 ± 0.016g, ANOVA F_1,42_ = 9.66; p = 0.003; small prawns: hungry = 0.004 ± 0.004g, satiated = 0.03 ± 0.004g, ANOVA F_1,43_ = 22.39; p < 0.001).

For Individual tasks, we used a total of 96 individuals (24 from each of the four size and hunger state treatments). For Group tasks, we aimed to use 14 groups of 16 individuals, with four individuals of each treatment per group. However, aggression within sets of prawns housed together prior to testing occasionally resulted in injury or death, causing some minor variation in final sample sizes between experimental treatments, and between spatial and manipulation tasks. For Group spatial tasks final sample sizes were: large, satiated = 55; large, hungry = 54; small, satiated = 56; small, hungry = 52. For Group manipulation tasks sample sizes were large, satiated = 53; large, hungry = 53; small, satiated = 54; small, hungry = 55.

### Experimental Set-up

To assess context-dependent variation in drivers of innovation, we conducted two different foraging tasks in both Individual and Group contexts. Each prawn took part in both the “spatial” and the “manipulation” tasks but was only tested in a single social context (either Individual or Group context). Once a prawn had participated in one task it was not used again 48 hours to reduce the likelihood of results being influenced by prior experience.

**Fig 1:**
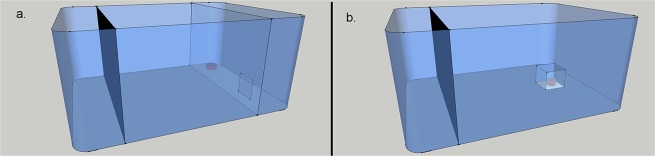
Experimental set up. The figure shows (a) the spatial task and (b) the manipulation task, with the opaque partition still in place (depicted by the black screen). In the spatial task, prawns had to go through the hole in the clear screen to gain access to the food reward (depicted as a red cube). In the manipulation task, a transparent box blocked access to the food rewards.

All trials were conducted in the morning between 9 and 12am. Trials were conducted in test tanks (measuring 30 x 40cm and containing 3 litres of fresh lab-produced saline water) and each test tank was used for a single trial per day. At the start of each trial, we used a net to transfer the individual or group of prawns into one end of a test tank, behind an opaque divider 5cm from the end of the tank. After 60 seconds, the opaque divider was removed, allowing the subject(s) access to the rest of the tank. The spatial task (Fig 1A), consisted of a clear divider 5cm from the far end of the tank, with a 3x3cm hole located 3cm from the side of the tank and 2cm from the bottom. The hole was large enough for even the biggest prawns to pass through with ease. Five blood worms were attached to a weight (small rock) with an elastic band and placed on the opposite side of the tank to the divider hole, 3cm from the side of the tank and 2.5cm from the end. Perforations in the divider allowed the diffusion of odour cues. To obtain the food, prawns had to take an indirect route to find the hole, rather than moving directly towards the food. In the manipulation task (Fig 1B), 5 blood worms were held in a fine netting parcel underneath a clear 2 x 2 x 2 cm plastic box with 0.5mm diameter perforations to allow the diffusion of odour cues. The box was placed 5cm from the opposite end of the tank to the divider and 9cm from either side of tank with only the bottom side left open, such that subjects had to flip over the box to obtain the food.

We conducted eight separate Individual prawn tasks per day, using four large and four small individuals, all of which were either satiated or food deprived (hunger state randomly allocated across days). Each prawn took part in both the manipulation and spatial task (task type randomly allocated across days), with two days between testing. Trials were terminated once the prawn obtained the food or when 300s had elapsed. For the Group tasks, we set up experimental groups of 16 prawns the day before each trial. Each group consisted of a randomly selected set of four individuals from each treatment, with treatments within each group being identifiable by their elastomer tags. The whole group of 16 was introduced into the test tank and we followed the same methods as for individual tasks, recording which individual prawn was first to complete the task. In between taking part in the two tasks, prawns were re-housed in identifiable sets of individuals of the same size and hunger state. The same groups of 16 prawns were used for spatial and manipulation tasks, with the order counterbalanced across groups.

### Data Scoring and Statistical Analysis

Experiments were recorded on a Panasonic HC-V110 EG-K camcorder. For the individual spatial tasks we noted how long it took the individual to reach the dividing barrier and whether it obtained food. For group spatial tasks we recorded the time, size and hunger state of the first prawn to obtain food in each group. For the individual manipulation tasks, we recorded the amount of time individuals spent manipulating the box (defined as physical contact with any part of the body) and, for successful individuals, the total time taken until they obtained food. We also noted the motor actions successful prawns used to extract food. For group tasks, we noted the timing, size, and hunger state of the first individual to reach the box, the first individual to obtain food and the motor technique used.

Statistical analyses were performed in R version 3.0.3 (The R Foundation for Statistical Computing, 2014). The two social contexts (Individual and Group) and the two task types (spatial and manipulation) were analysed separately. For Individual tasks, we used Generalised Linear Models with a binomial error structure indicating whether or not each prawn solved the task (1,0), with size (large/small) and hunger state (satiated/hungry) fitted as response terms. Task order (whether the task was the first or second each prawn performed) was fitted as an additional explanatory term. The limited variation in species (94% of prawns were *P*. *elegans*) precluded including species as an explanatory term, but informal analyses revealed no behavioural differences between the two species.

To determine whether the treatments differed in their motivation to move towards the food in the spatial task, we used a GLM with time taken to reach the barrier (in seconds) as the response term and size, hunger and task order as explanatory terms. Data were fitted to a quasi-poisson distribution as they showed over-dispersion relative to a Poisson distribution. For the manipulation task, we used GLMs with quasipoisson error structure to examine the effects of size, hunger and task order on the time to reach the box and the time spent manipulating the box.

To examine the factors influencing success in Group tasks, we used GLMMs with a binomial error structure (1,0) indicating whether each treatment category was successful (i.e. a member of the group was the first prawn to complete the task). Group identity was fitted as a random term, with size, hunger and task order fitted as explanatory terms. To account for the slight variation in prawn numbers, the number of prawns in treatment category in each group was fitted as an additional covariate.

## Results

### Individual Spatial Tasks

**Fig 2:**
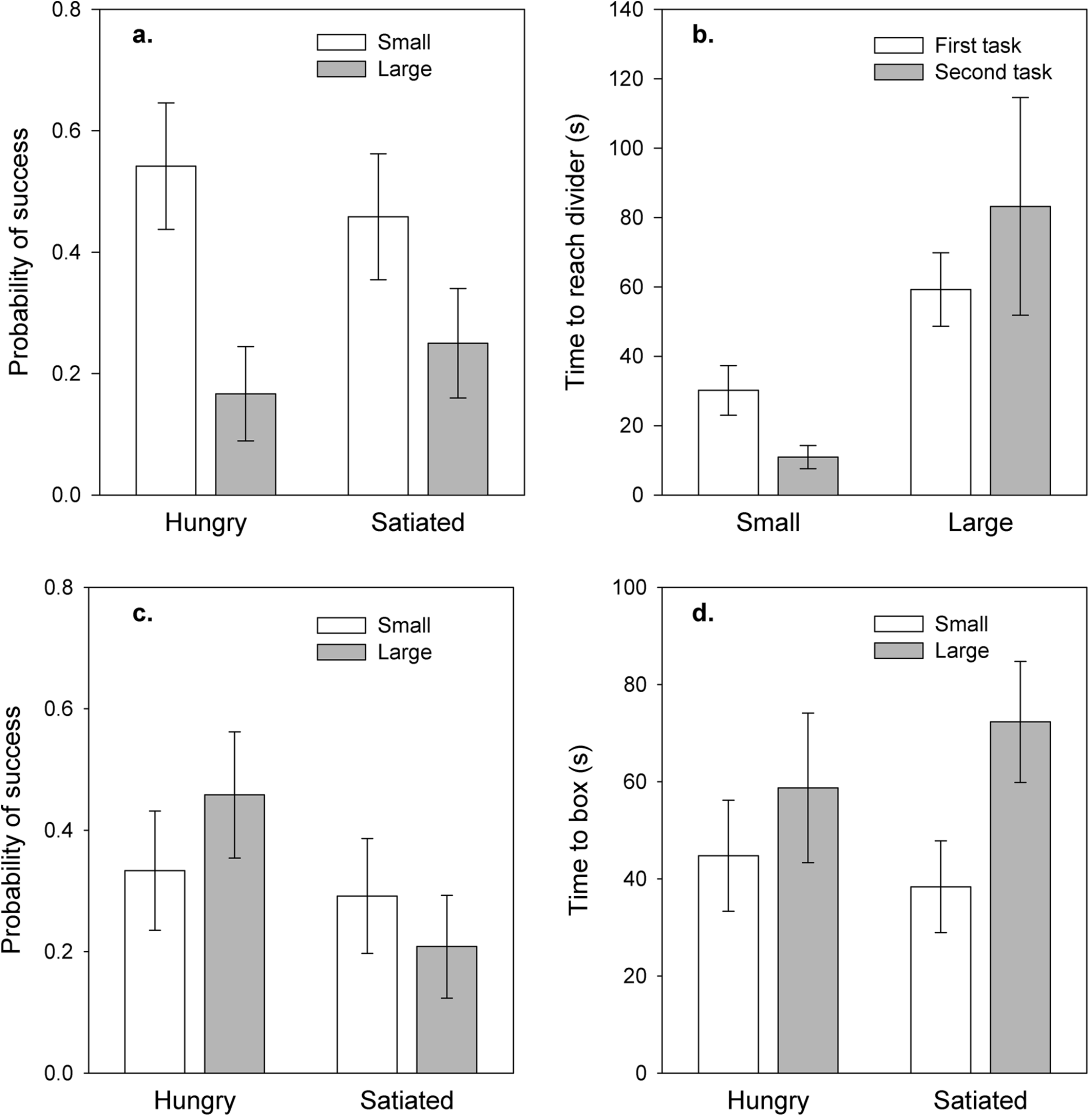
Factors influencing performance in single prawn tasks. Spatial tasks: (a) probability of success and (b) time to reach the divider. Manipulation tasks (c) probability of success and (d) time to reach the box. Bars show means ± SE from raw data.

Small prawns were significantly more likely than large prawns to succeed in obtaining food (GLM: χ^2^ = 8.52, df = 1, *p* = 0.004; Fig 2A): 24 out of 48 small individuals (= 50%) were successful, compared to 10 out of 48 large prawns (= 21%). Hunger state had no effect on the probability of success, with half the successful individuals being hungry (N = 17) and half being satiated (GLM: χ^2^ = 0.00, df = 1, *p* = 1), and there was no significant interaction between size and hunger (χ^2^ = 0.83, df = 1, *p* = 0.36; Fig 2A). Task order had no significant influence on success (χ^2^ = 0.067, df = 1, *p* = 0.796).

Six prawns, all of them large, failed to reach the central divider during the test. Among the remaining 91 prawns, small individuals reached the divider significantly faster than large prawns and were particularly fast if they had previously taken part in the manipulation task (GLM; effect of size: *F*_1_ = 16.11, *p* < 0.001; size*task order interaction: *F*_1_ = 4.43, *p* = 0.038; Fig 2B). Hungry and satiated individuals did not differ in time taken to reach the barrier (effect of hunger: *F*_1_ = 0.77, *p* = 0.38; hunger*size: *F*_1_ = 0.94, *p* = 0.33).

### Individual Manipulation Tasks

Neither size nor hunger had a significant effect on the probability of success (GLM: size: χ^2^ = 0.048, df = 1, *p* = 0.827; hunger: χ^2^ = 2.31, df = 1, *p* = 0.123; size*hunger: χ^2^ = 1.18, df = 1, *p* = 0.278; Fig 2C), and there was no significant effect of task order (χ^2^ = 1.88, df = 1, *p* = 0.171). Of the 31 successful individuals 16 were large (of which 11 were hungry) and 15 were small (of which eight were hungry). A further 52 individuals interacted with the box but did not succeed in obtaining food. Successful and unsuccessful individuals did not differ in the time they spent manipulating the box (Mann Whitney U test: U = 701.5; *p* = 0.328). However, small prawns were significantly faster to reach the box than large prawns (GLM: *F*_1_ = 4.62, *p* = 0.035; Fig 2D) and spent longer manipulating the box (GLM: *F*_1_ = 6.14, *p* = 0.015). Hunger state did not influence the time to reach the box (GLM hunger: *F*_1_ = 0.08, *p* = 0.78; hunger*size: *F*_1_ = 0.70, *p* = 0.41) or the time spent manipulating it (GLM hunger: *F*_1_ = 0.00, *p* = 0.95; hunger*size: *F*_1_ = 0.04, *p* = 0.84). Task order did not influence the time to reach the box (*F*_1_ = 0.055, *p* = 0.458) but prawns spent more time manipulating the box if the manipulation task was their second task (*F*_1_ = 6.54, *p* = 0.012).

Among the successful individuals, small and large prawns tended to use different motor techniques to access food: 14 of the 16 large prawns flipped the box over, whereas all 15 small prawns reached through the small perforations in the box to pull food out (Chi square test comparing the distribution of techniques between the two size categories: χ^2^ = 23.93, df = 1, *p* < 0.001). Hunger state had no influence on the technique used (Chi square test: χ^2^ = 0.10, df = 1, *p* < 0.756).

### Between Individual Tasks

There was no indication that prawns that succeeded in one task were particularly likely to succeed in the other. In total, 49 individuals succeeded in one task and not the other. Only eight individuals were successful in both tasks, a number that did not differ from chance expectations (Chi square test: χ^2^ = 0.53; df = 1, *p* = 0.468). Moreover, among these eight individuals there was no significant correlation between the time taken to solve the two tasks (Spearman’s rank test: *t* = 0.73; df = 1, *p* = 0.286).

### Group Spatial Tasks

**Fig 3:**
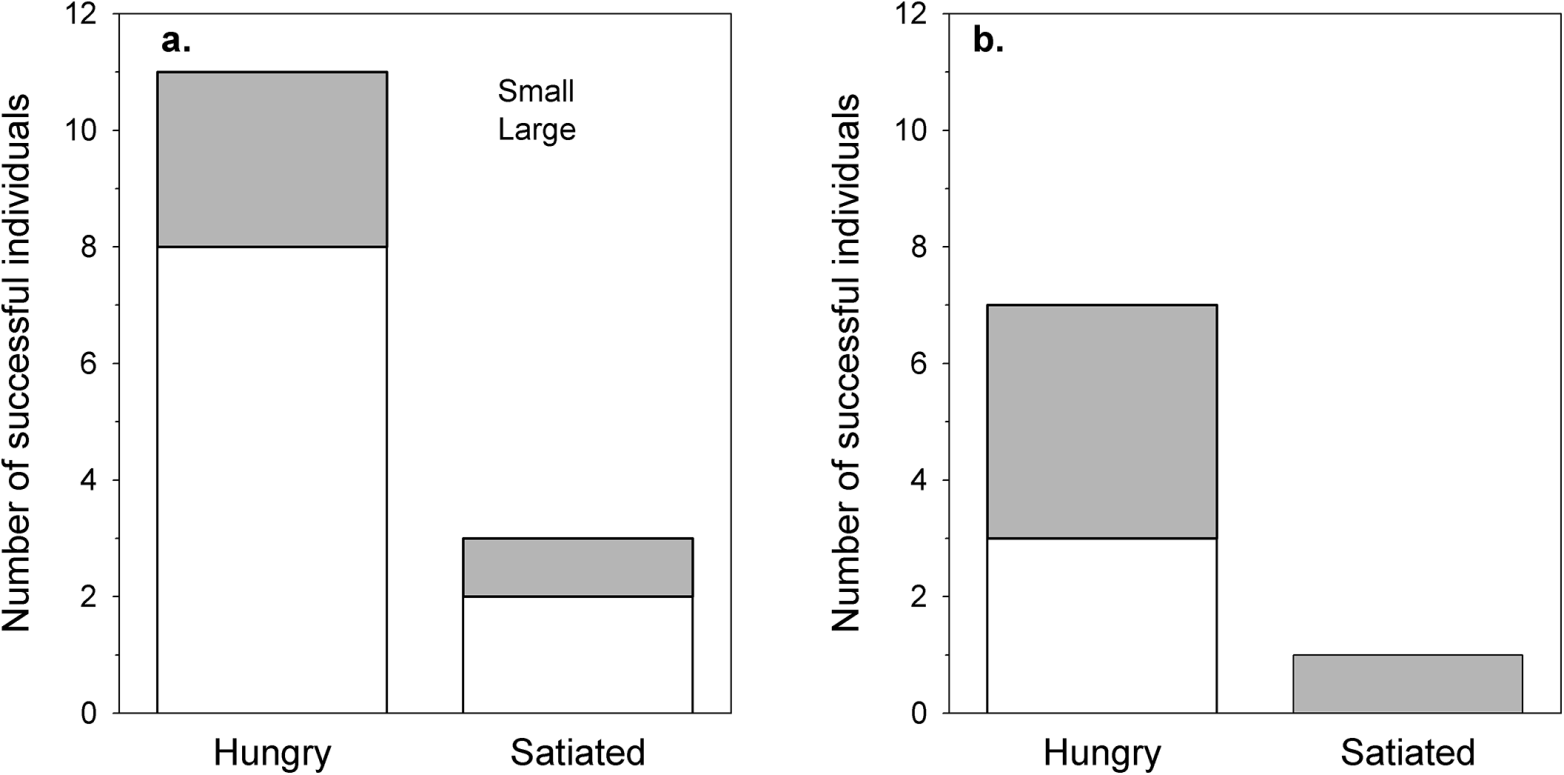
Number of successful individuals of each size and hunger level in group tasks. (a) Spatial tasks; (b) manipulation tasks. White bars = small prawns; grey bars = large prawns.

Hungry individuals were disproportionately likely to be the first in the group to obtain food (GLMM χ^2^ = 5.08, df = 1, *p* = 0.029): of the 14 successful individuals, 11 were hungry (Fig 3A). Although 10 small individuals completed the task, compared to only 4 large, this effect did not reach statistical significance (size: χ^2^ = 3.55, df = 1, *p* = 0.065; size*hunger: χ^2^ = 0.26, df = 1, *p* = 0.609). Task order had no significant effect on success (χ^2^ = 0.01, df = 1, *p* = 0.938).

### Group Manipulation Tasks

Across the 14 groups (215 individuals), only eight individuals completed the task, with seven of these being hungry (GLMM, effect of hunger: χ^2^ = 4.10, df = 1, *p* = 0.048). There was no significant effect of size (χ^2^ = 0.42, df = 1, *p* = 0.519), no interaction between size and hunger (χ^2^ = 0.03, df = 1, *p* = 0.868; Fig 3B), and no effect of task order (χ^2^ = 0.01, df = 1, *p* = 0.935). All of the successful large prawns obtained the food by flipping over the box, whereas two of the three successful small prawns pulled food out of the perforations in the box. As the elastomer tags identified prawns in Group tests by their experimental treatment, rather than as individuals, we were not able to test formally whether individuals that succeeded in one Group task (spatial or manipulation) were more likely to succeed in the other.

## Discussion

Behavioural innovation is thought to play an important role in enabling animals to cope with environmental change [2,4]. Research on animal innovation has focused on terrestrial and freshwater vertebrates [6,28], but few animals face environmental variation as extreme as those living in littoral zones, where physical and social conditions change dramatically from moment to moment. Here we show that the factors that drive rock pool prawns to innovate differ depending on the conditions in which they find themselves. When tested alone in the spatial task, small prawns were significantly more likely to succeed than large individuals, irrespective of their hunger level. Size had no significant effect on success in the spatial task, but smaller prawns were faster to reach the parcel containing food, while hunger again had no effect. In contrast, hunger was the main driver of innovation when prawns were tested in groups. In both the spatial and manipulation tasks, hungry individuals were disproportionately likely to succeed, but size had no effect.

In the spatial task, small prawns were more than twice as likely to succeed in locating the food compared to their large counterparts and were significantly faster to reach the barrier. Small individuals reached the barrier particularly quickly if they had previously taken part in the manipulation task, suggesting that prior experience in testing tanks may have heightened motivation or reduced wariness. Contrary to our predictions, food deprivation had no influence on innovation, suggesting that successful prawns were not driven by the need to address immediate deficits in their energy budgets. There are a number of possible explanations for the effect of body size. The 3 x 3 cm hole in the apparatus was large enough for even the fattest prawn to pass through easily, so the result is unlikely to be an artefact of task design. A more likely possibility is that small individuals, which are likely to be at a disadvantage in scramble competition, are driven to seek new ways of finding food when there is no risk of having their gains stolen by larger competitors. Smaller individuals may also be less conspicuous and less valuable to predators [29], making them more willing to take the risks of exploring to discover food rewards. Small size may also be linked to other variables that influence innovatory propensities. For instance, prawns show indeterminate growth [30], so smaller individuals are also likely to be younger. Youth is often associated with increased exploration, reduced neophobia and heightened persistence, and in some species these propensities appear to be linked to increased success in novel problem-solving tasks (see [6] for a review). Finally, *Palaemon* prawns are sexually dimorphic, with males generally being smaller than females, and populations tend to show male biased sex ratios [28]. Recent work on *P*. *elegans* suggests that males are typically bolder and more active than females [31], perhaps as a result of the need to seek mates. We were unable to sex prawns in our study, but it is possible that these proclivities may also predispose males to innovate, generating the apparent effect of small size in our results.

Small size was also associated with speed in reaching the food parcel in the manipulation task, although here smaller individuals were not more likely to succeed in extracting the resource. The physical nature of the task is likely to have made it particularly difficult for smaller, less powerful individuals, meaning that they had to spend more time manipulating the box before succeeding. Thus, for small individuals at least, persistence may be crucial for innovation, mirroring findings in a number of vertebrate species (e.g. [9,14,15,32–34]). Motivation to persist may itself be affected by prior experience, as prawns spent more time manipulating the box if the manipulation task was their second task. In our experiment, size differences also led to differences in technique, with the larger individuals preferring to flip the box over while the smaller ones, who may not have had the strength to do so, tended to employ a more laborious method of reaching through the tiny perforations in the box to extract food. Thus size may not only affect whether or not innovative behaviour occurs, but also the motor actions it employs. Our finding that, in Individual tests, prawns that succeeded in one task were not disproportionately likely to succeed in the other highlights the fact that different attributes may favour innovative behaviour in different contexts.

This conclusion is further supported by the group tasks, where size had no effect and hunger was the driving factor leading individuals to innovate. In both the spatial and manipulation group tasks, food deprived individuals were disproportionately likely to succeed, irrespective of their size. Small individuals may derive little benefit from investing in novel behaviours if there is a high risk that any food they discover will be stolen. Consequently, in group contexts the effects of small body size on individuals’ motivation to seek new ways of obtaining food are diminished, and innovation instead appears to be driven by immediate nutritional needs. Similar effects of hunger have been reported in studies of guppies, but studies of birds and mammals have failed to find clear effects of energetic state. However, this may reflect a reliance on loose proxies of current state (e.g. fat scores [14]; body weight and foraging efficiency [9]) rather than the lack of an effect per se (but see [35]). Future studies incorporating strict experimental control over food intake may help reveal hidden effects of energetic state on innovation in group-living animals [6].

Our finding that small and hungry prawns tend to be the innovators is broadly in accordance with the necessity drives innovation hypothesis. However, our results suggest that the hypothesis is overly simplistic and lacks clear explanatory power. For many animals, as for our prawns, social conditions may vary substantially over time. Many birds, for example, form large flocks during the winter but are relatively solitary and territorial at other times of year. Similarly, animals living in fission-fusion societies will find themselves in groups of varying sizes, and dispersing individuals will have periods of relative solitude when seeking new territories and mating opportunities. Thus, it seems unlikely that any given factor or set of factors will explain variation in innovation in all circumstances. For instance, a number of studies have assumed that innovative individuals are particularly cognitively adept [2,36–38]. Our work cautions against there being clear causal links between individual cognitive abilities and innovation as we found no evidence for individual consistency between tasks (see also [9,39,40]). We suggest that important advances in our understanding of variation in innovatory propensities may be made by considering the potential risks and benefits associated with investment in exploration and the production of novel behavioural patterns under different conditions. If we are to understand the factors that drive innovation, and subsequently make available novel information that has the potential to spread through groups, we must begin to examine how and why different phenotypic and state-related factors lead individuals to innovate across contexts.
